# Supplementary material for: Association of Dietary Total Antioxidant Capacity with Bone Mass and Osteoporosis Risk in Korean Women: Analysis of the Korea National Health and Nutrition Examination Survey 2008–2011
Source: Nutrients. 2021 Mar 31;13(4):1149. doi: 10.3390/nu13041149 (PMC8065953; doi:10.3390/nu13041149)
Supplement: Supplementary file 1 [file nutrients-13-01149-s001.zip › Dietary Total Antioxidant Capacity Calculator instruction.docx]

**Dietary Total Antioxidant Capacity Calculator instruction**

This program named ‘Dietary Total Antioxidant Capacity Calculator (DTACC)’ calculates the daily dietary Total Antioxidant Capacity (TAC) for each ID using amount of daily intake foods and contents of antioxidant nutrients per 100g of foods. The amount of daily intake foods data was collected by the 24-hour recall method conducted by the Korea National Health and Nutrition Examination Survey (KNHANES) 2008-2011. The dietary TAC was obtained using the Equation 1 for calculating the theoretical TAC [1]. Daily dietary TAC was calculated as the sum of all dietary TAC that consumed during 24-hour each person (Equation 2). Processing code is written in Python V. 3.8.3 (64-bit) using the Python libraries Openpyxl V.3.0.4 and Pandas V.1.1.0.

Equation 1 :

$Theoretical TAC=\sum\left( antioxidant content\frac{mg}{100g}\times antioxidant capacity\frac{mg VCE}{100g} \right)$ **(1)**

Equation 2 :

$Daily dietary TAC=\sum(Daily intake food amount \times theoretical TAC (per 1g of food ))$ **(2)**

The DTACC required an antioxidant data base (DB) file and KNHANES files to calculate daily dietary TAC. The antioxidant DB file was characterized by the following variables: ‘N_FNAME’, ‘N_FNAME3’, ‘Sort’, ‘Beta-Carotene (microgram)’, ‘Alpha-Carotene (mg)’, ‘Beta-Cryptoxanthin (mg)’, ‘Lycopene (mg)’, ‘Lutein (mg)’, ‘Zeaxanthin (mg)’, ‘Retinol (microgram)’, ‘Ascorbic acid (mg)’, ‘Alpha-Tocopherol (mg)’, ‘Gamma-Tocopherol (mg)’, ‘Quercetin (mg)’, ‘Kampferol (mg)’, ‘Myricetin (mg)’, ‘Isorhamnetin (mg)’, ‘Luteolin (mg)’, ‘Apigenin (mg)’, ‘Hesperetin (mg)’, ‘Naringenin (mg)’, ‘Eriodictyol (mg)’, ‘(+)-Catechin (mg)’, ‘(+)-Gallocatechin (mg)’, ‘(-)-Epicatechin (mg)’, ‘(-)-Epigallocatechin (mg)’, ‘(-)-Epicatechin 3-gallate (mg)’, ‘(-)-Epigallocatechin 3-gallate (mg)’, ‘Theaflavin (mg)’, ‘Theaflavin 3-gallate (mg)’, ‘Theaflavin 3’-gallate (mg)’, ‘Theaflavin 3,3’-digallate (mg)’, ‘Cyanidin (mg)’, ‘Delphinidin (mg)’, ‘Malvidin (mg)’, ‘Pelargonidin (mg)’, ‘Peonidin (mg)’, ‘Petunidin (mg)’, ‘Daidzain (mg)’, ‘Genistein (mg)’, ‘Glycitein (mg)’, ‘Biochanin A (mg)’, ‘Formononetin (mg)’, ‘Procyanidin (mg)’, and ‘Cinnamtannin B1 (mg)’. The first food name was inputted in the ‘N_FNAME’ column, and the third food name was inputted in the ‘N_FNAME3’ column, and one of ‘0’ or ‘1’ or ‘e’ was inputted in the ‘Sort’ column. The subsequent 42 columns mean 42 dietary antioxidants contained per 100g of each food; the blanks were calculated to be zero. ‘0’ in the ‘Sort’ column means that the nutrient amount of the first food was inputted, ‘1’ means that the nutrient amount ​​of the third food was inputted, and ‘e’ mean the excluded food. The file name was ‘dtacdb.xlsx’, and the sheet name was ‘dtacdb’. The KNHAMES files are sorted in the order of ‘ID’, and then the variables are extracted including ‘ID’, ‘N_FNAME’, ‘N_FNAME3’, ‘NF_INTK’, ‘NF_INTK3’, ‘NF_RETIN’, ‘NF_VITC’. The sheet name was labeled the same name as the file name and was saved in the Excel file form.

The program is divided into four parts: the first part is a part that searching the antioxidant DB file and a KNHANES data file and identifying the format of the files. The second part calculates the antioxidant capacity of nutrients contained in each food by the antioxidant DB file. The third part, reading the KNHANES file, calculates antioxidant capacity for each food item (‘outputdetail_(input file name)’). The last part calculates daily dietary TAC by adding the antioxidant capacity consumed by each ID (‘output_(input file name)’).

**First part**

The program checks whether the antioxidant DB file exists in the same folder of this program; and then, it affirms the name of the sheet to be used. If the ‘dtacdb.xlsx’ file has not existed in the same folder of this program, it would create the file in the example form. If the antioxidant DB file met the conditions, the program should affirm the file name of the KNHANES file to calculate and affirm that the file exists in the parent folder of this program. In the same way, the program checks the KNHANES file and would affirm the name of the sheet to be used.

**Second part**

The program receives the antioxidant DB file and multiplies the amount of nutrients contained per gram of each food by the antioxidant capacity of each nutrient. In the form of a dictionary, match the antioxidant capacity values of nutrients contained per gram of the food and the values ​​of the ‘Sort’ line according to the primary food name (‘N_FNAME’).

**Third part**

The program receives the KNHANES file and find the food name in the dictionary created in the second part. It calculates the TAC value of each food by multiplies amount of the food intake according to the ‘Sort’ value, the nutrient content, and the antioxidant activity of each nutrient (Equation (1)). Excluded foods are labeled ‘Excluded food’ and antioxidant capacity values of the food are calculated as zero and foods that were not in the antioxidant DB file are tagged ‘Missing data’. It output the values ​in the ‘outputdetail_(input file name).xlsx’ file to review which food is the problem when there is an error in the result of the fourth part.

**Fourth part**

The program adds all the TAC values with the same ID in a result of third part to calculate a TAC value consumed per day for each ID (Equation (2)). When the program found ‘Missing data’, it tagged ‘There's missing data here’. Output the completed results to the ‘output_(input file name).xlsx’ file.

1. Floegel A, Kim DO, Chung SJ, Song WO, Fernandez ML, Bruno RS, Koo SI, Chun OK (2010) Development and validation of an algorithm to establish a total antioxidant capacity database of the US diet. Int J Food Sci Nutr 61 (6):600-623. doi:10.3109/09637481003670816
